# Supplementary material for: SHMT2 Induces Stemness and Progression of Head and Neck Cancer
Source: Int J Mol Sci. 2022 Aug 26;23(17):9714. doi: 10.3390/ijms23179714 (PMC9456418; doi:10.3390/ijms23179714)
Supplement: Supplementary file 1 [file ijms-23-09714-s001.zip › ijms-1859532-supplementary.pdf]

Supplementary Figure S1

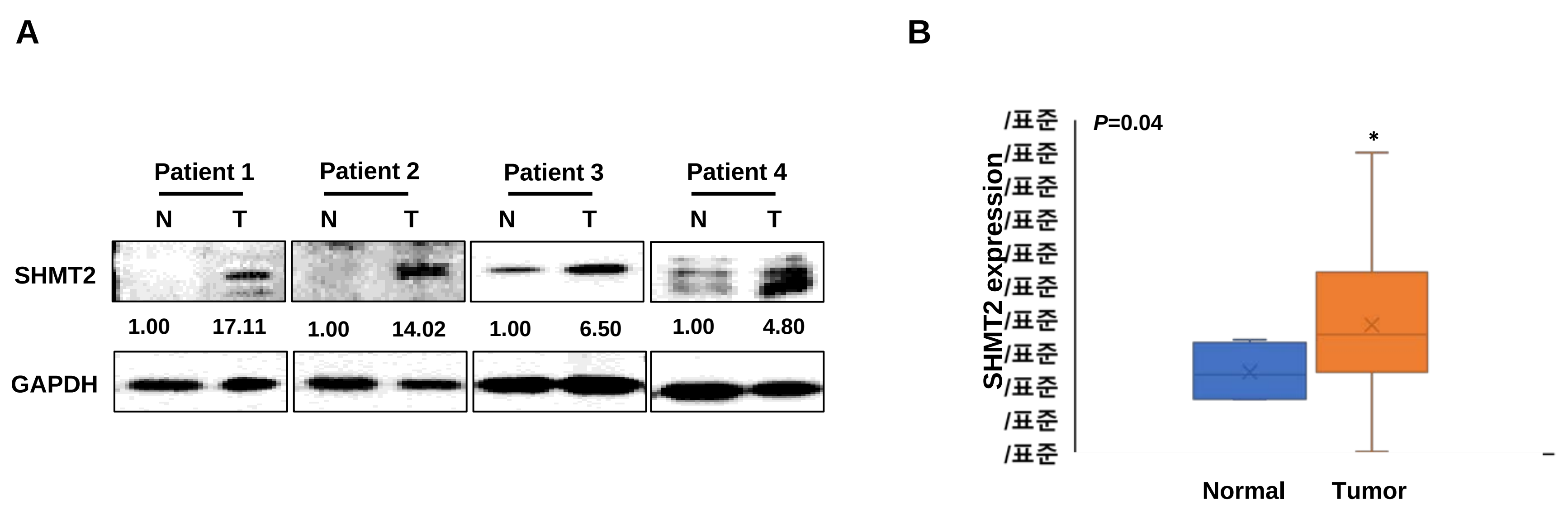


**Figure S1. SHMT2 was overexpressed in HNC patients from CNUH.** (A) Four paired tissue samples obtained from the same patients with HNC were examined by western blot analysis. (B) SHMT2 was overexpressed in 43 HNC tissues compared to four normal tissues in the CNUH cohort.

**Supplementary Figure S2**

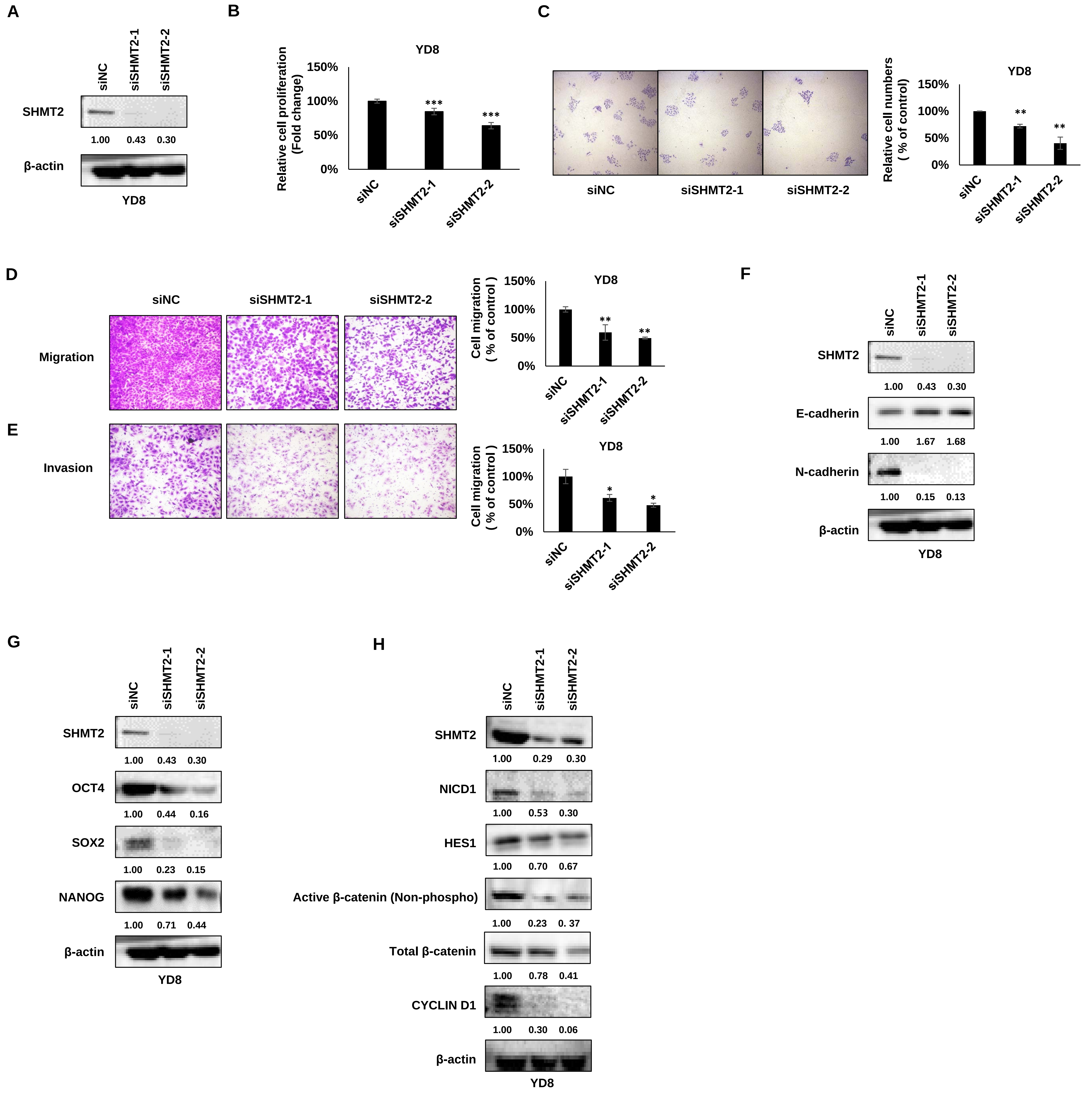


**Figure S2.** ***SHMT2*** **played an oncogenic role and regulated the epithelial–mesenchymal transition (EMT) in YD8 cells.** YD8 cells were transiently transfected with a SHMT2 siRNA and a negative control siRNA. (A, B) After transfection, the levels of SHMT2 were detected by Western blots and cell proliferation was analyzed using the WST-1 assay. (C) Representative images of colony formation assays from SHMT2 siRNA-transfected cell line and quantitative analysis of colony formation assays. (D, E) After transfection, the cells were allowed to migrate for 24 hours in Transwell chambers (cell migration) or for 48 hours in chambers coated with Matrigel (cell invasion) (F) Differences in the expression of EMT markers after transfection were detected by Western blotting. (G) The protein expression of OCT4, SOX2, and NANOG was analyzed by Western blotting. (H) Western blot analysis of Notch and Wnt pathway-related proteins with SHMT2 knockdown in YD8 cells. Data were presented as mean ± SD of three independent experiments. Differences were considered relevant at $p < 0.05$ (* $p < 0.05$, ** $p < 0.01$, *** $p < 0.001$).

Supplementary Figure S3

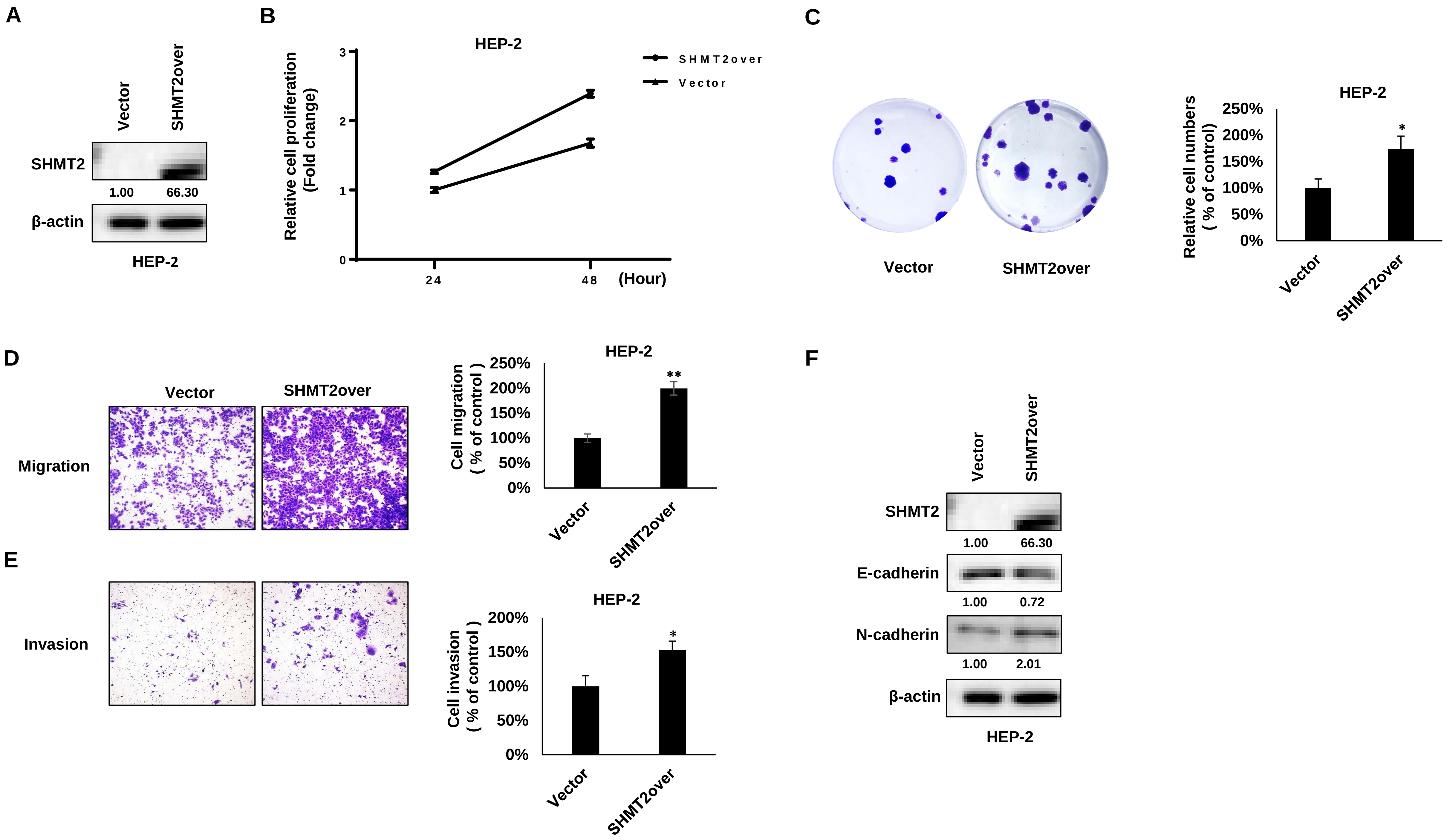


**Figure S3. SHMT2 overexpression increased cell proliferation, migration, and invasion in HEP-2 cells.** (A-C) Cell proliferation and colony formation were assessed in HEP-2 cells that were transiently transfected with a myc-SHMT2 vector and a negative control vector. (A) The levels of SHMT2 were detected by western blots. (B) The relative number of viable cells was significantly increased with SHMT2 overexpression compared to the control after 24 and 48 hours of incubation. (C) Colony formation was also increased in SHMT2-overexpressing cells. (D, E) After transfection, the cells were allowed to migrate for 24 hours in Transwell chambers (cell migration) or for 48 hours in chambers coated with Matrigel (cell invasion). Magnification, ×40. (F) Differences in the expression of epithelial-mesenchymal transition markers in HEP-2 cells transfected with the myc-SHMT2 vector and the negative control vector were detected by western blotting. Differences were considered relevant at $p < 0.05$ (* $p < 0.05$, ** $p < 0.01$, *** $p < 0.001$). All experiments were repeated at least three times.

**Supplementary Figure S4**

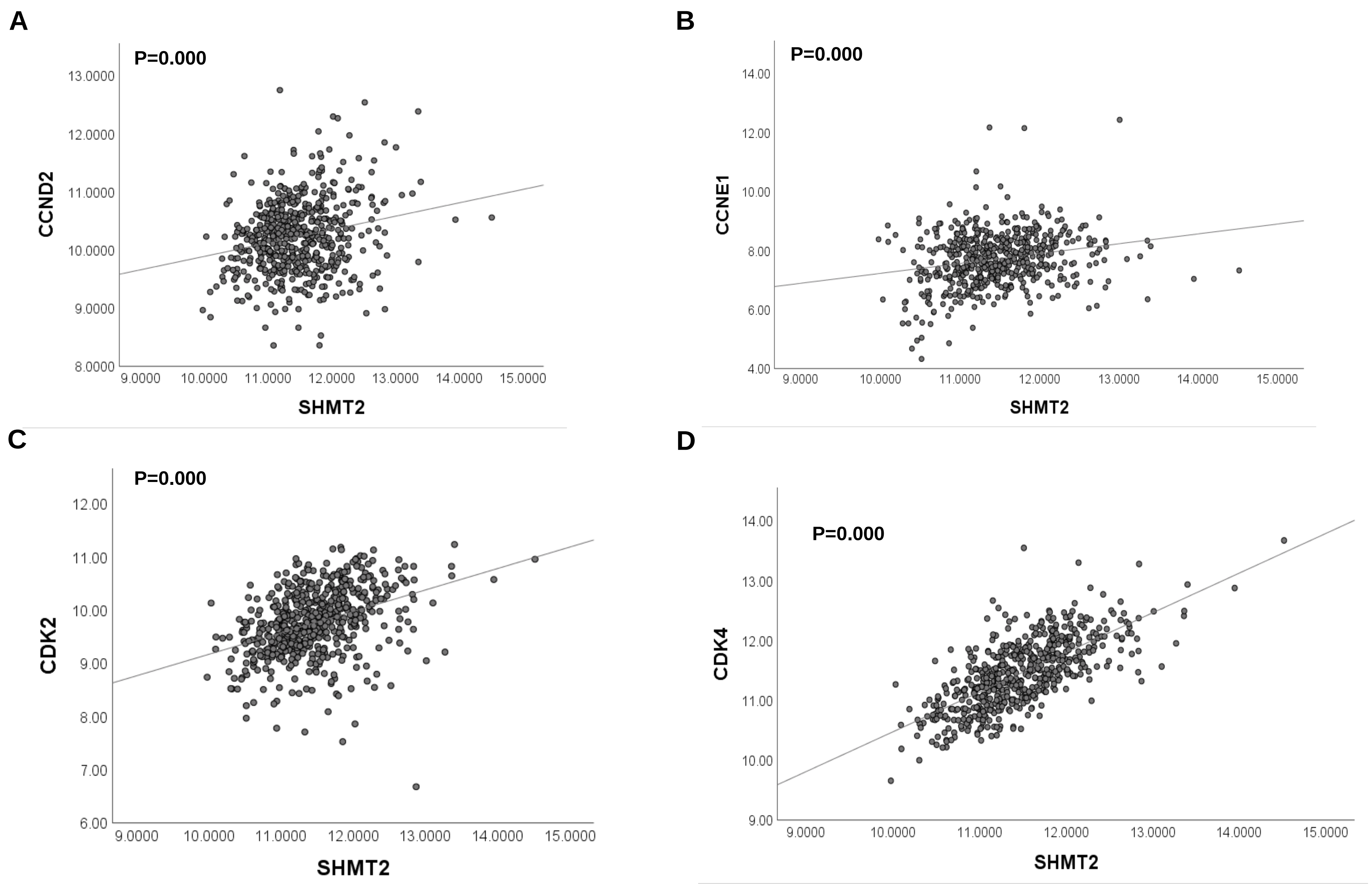


**Figure S4. Positive correlations between *SHMT2* and cell cycle-related genes in the TCGA database.** (A-D) Pearson correlation coefficients were calculated to verify the correlation between *SHMT2* and *CCND2*, *CCNE1*, CDK2, CDK4. Differences were considered relevant at $p < 0.05$.

**Supplementary Figure S5**

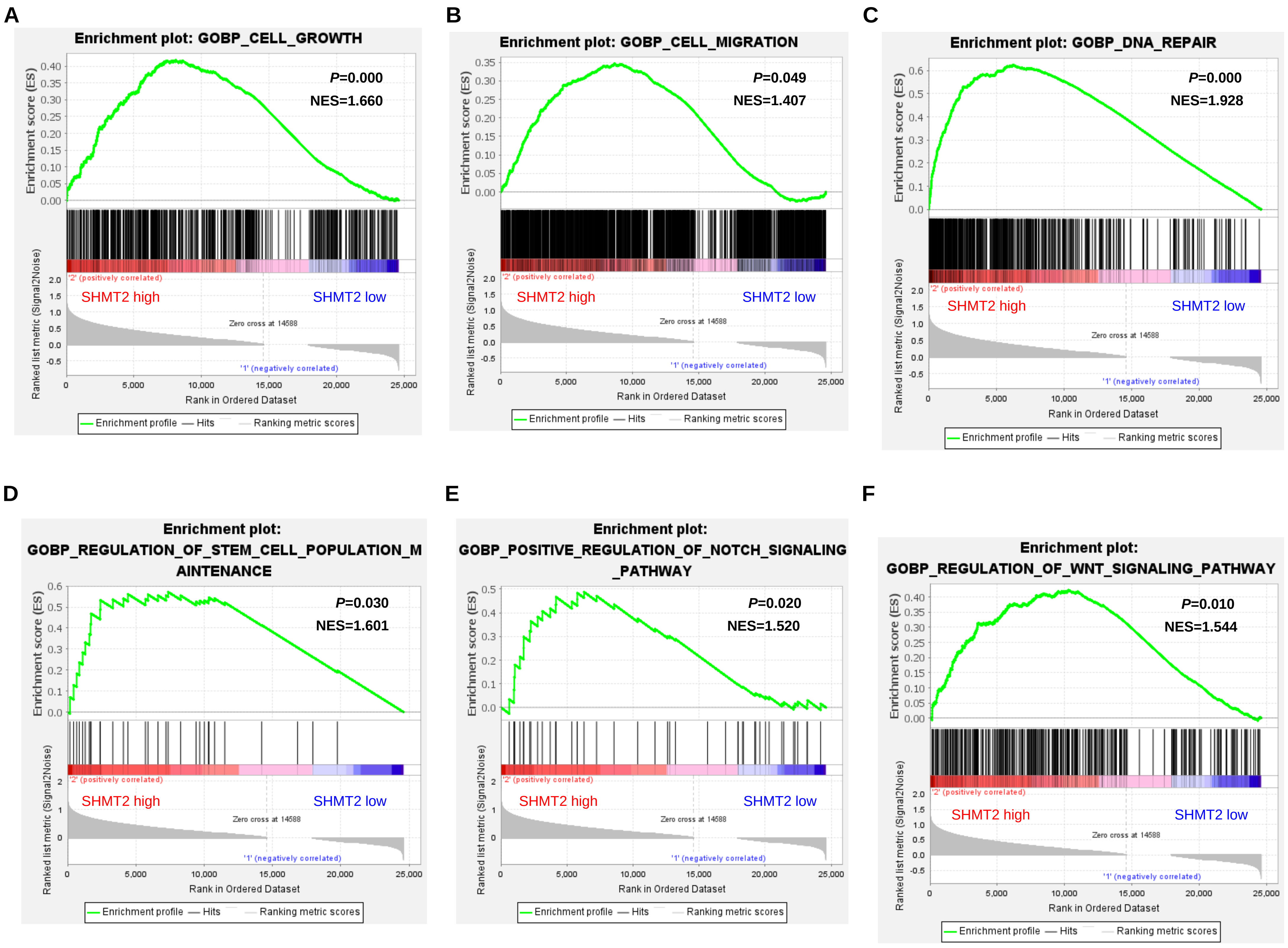


**Figure S5. Gene set enrichment analysis showed that *SHMT2* is associated with tumor progression in HNC patients from CNUH.** (A-F) Cell growth, cell migration, DNA repair, stem cell maintenance, and Notch and Wnt signaling pathway-related gene sets were enriched in HNC patients with high *SHMT2* expression in the CNUH database. Differences were considered significant at $p < 0.05$.

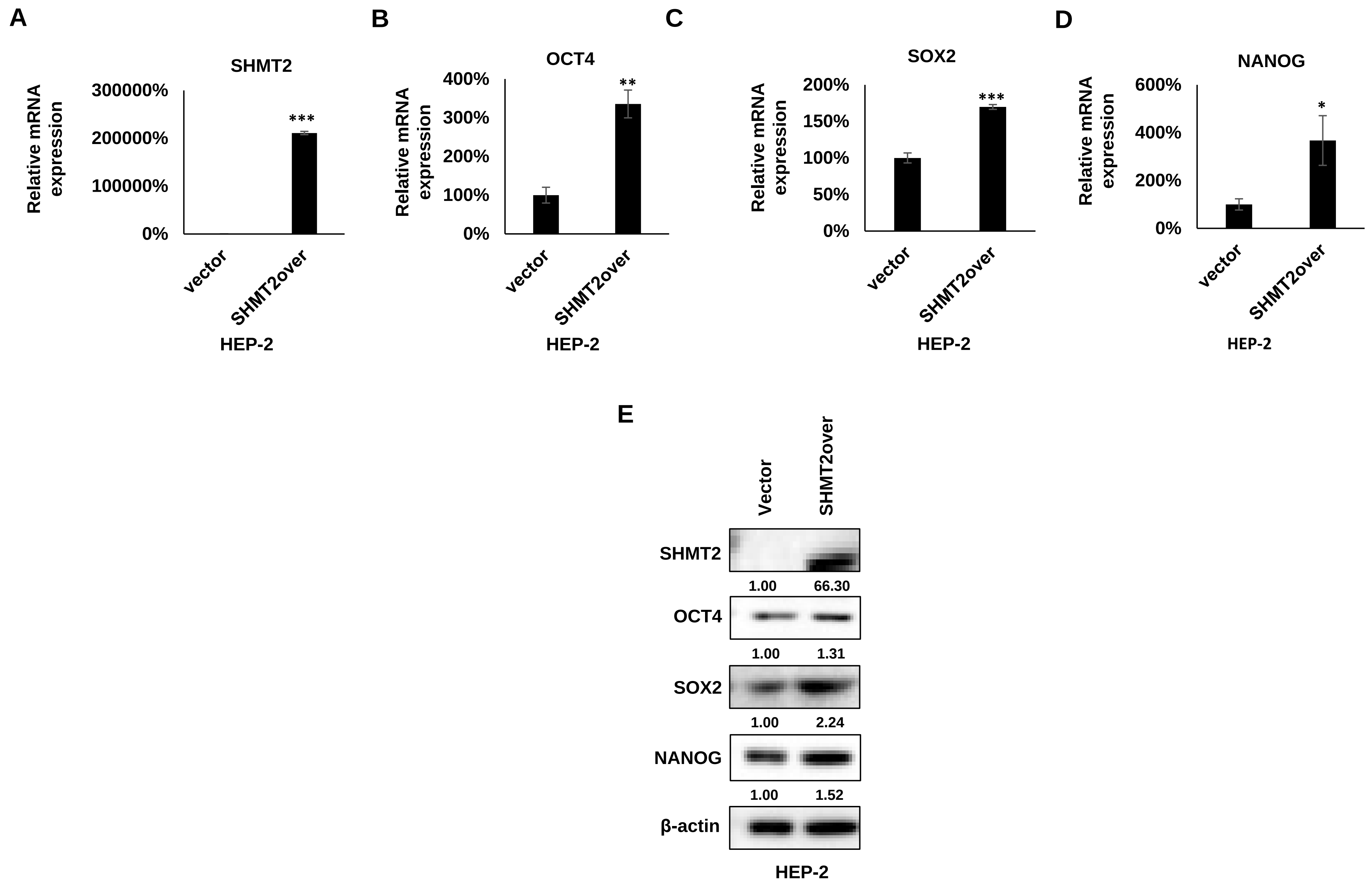


**Figure S6. *SHMT2* overexpression increased stemness markers in the HEP-2 cell line.** (A-D) Compared with the control group, *SHMT2* mRNA expression was significantly increased in cells transfected with the SHMT2-myc vector compared to those transfected with the negative control vector; the expression of *OCT4*, *SOX2*, and *NANOG* was also significantly increased. (E) The protein expression of OCT4, SOX2, and NANOG was significantly increased in SHMT2-myc transfected HEP-2 cells. Differences were considered relevant at $p < 0.05$ (* $p < 0.05$, ** $p < 0.01$, *** $p < 0.001$). All experiments were repeated at least three times.
